# Supplementary material for: Local-scale topoclimate effects on treeline elevations: a country-wide investigation of New Zealand’s southern beech treelines
Source: PeerJ. 2015 Oct 22;3:e1334. doi: 10.7717/peerj.1334 (PMC4627911; doi:10.7717/peerj.1334)
Supplement: Table S1 [file peerj-03-1334-s001.docx]

**SUPPLEMENTARY MATERIAL**

Table S1. Descriptive statistics for 17 explanatory variables used in random forest modelling.

| Explanatory factors | Minimum | Median | Mean | Maximum |
| --- | --- | --- | --- | --- |
| Landform |  |  |  |  |
| Slope (%) | 2.80 | 60.48 | 62.02 | 200.30 |
| Curvature index | -3.35 | 0.06 | 0.05 | 2.89 |
| Topoclimate |  |  |  |  |
| January frost index | 0.00 | 1353.00 | 1431.70 | 4016.50 |
| January photoinhibition index | 264.90 | 785.10 | 800.90 | 1494.60 |
| January desiccation index | 36.08 | 224.58 | 245.15 | 942.57 |
| January insolation | 129880 | 222500 | 220512 | 288662 |
| July frost index | 0.00 | 1162.50 | 1189.40 | 3151.00 |
| July photoinhibition index | 27.19 | 250.28 | 262.60 | 692.63 |
| July desiccation index | 36.74 | 155.44 | 188.18 | 629.13 |
| July insolation | 7585 | 59936 | 58740 | 135331 |
| Regional |  |  |  |  |
| Mean growing season temperature (°C) | 9.67 | 11.32 | 11.33 | 13.95 |
| Mean winter temperature (°C) | -0.49 | 2.39 | 2.42 | 5.72 |
| Mountain mass index (km^2^) | 0.00 | 15.24 | 15.03 | 40.98 |
| Total annual precipitation (mm) | 1231 | 3108 | 3852 | 8759 |
| Earthquake intensity index | 0.33 | 0.54 | 0.52 | 0.65 |
| Erosion index | 11.00 | 33.00 | 39.92 | 136.00 |
| Topographic variation (m) | 157.70 | 292.00 | 291.20 | 498.20 |
